# Turning-Off Signaling by Siglecs, Selectins, and Galectins: Chemical Inhibition of Glycan-Dependent Interactions in Cancer

**DOI:** 10.3389/fonc.2016.00109

**Published:** 2016-05-13

**Authors:** Alejandro J. Cagnoni, Juan M. Pérez Sáez, Gabriel A. Rabinovich, Karina V. Mariño

**Affiliations:** ^1^Laboratorio de Glicómica Funcional y Molecular, Instituto de Biología y Medicina Experimental (IBYME), Consejo Nacional de Investigaciones Científicas y Técnicas (CONICET), Buenos Aires, Argentina; ^2^Laboratorio de Inmunopatología, Instituto de Biología y Medicina Experimental (IBYME), Consejo Nacional de Investigaciones Científicas y Técnicas (CONICET), Buenos Aires, Argentina; ^3^Facultad de Ciencias Exactas y Naturales, Universidad de Buenos Aires, Buenos Aires, Argentina

**Keywords:** glycans, cancer, siglecs, C-type lectins, selectins, galectins

## Abstract

Aberrant glycosylation, a common feature associated with malignancy, has been implicated in important events during cancer progression. Our understanding of the role of glycans in cancer has grown exponentially in the last few years, concurrent with important advances in glycomics and glycoproteomic technologies, paving the way for the validation of a number of glycan structures as potential glycobiomarkers. However, the molecular bases underlying cancer-associated glycan modifications are still far from understood. Glycans exhibit a natural heterogeneity, crucial for their diverse functional roles as specific carriers of biologically relevant information. This information is decoded by families of proteins named lectins, including sialic acid-binding immunoglobulin (Ig)-like lectins (siglecs), C-type lectin receptors (CLRs), and galectins. Siglecs are primarily expressed on the surface of immune cells and differentially control innate and adaptive immune responses. Among CLRs, selectins are a family of cell adhesion molecules that mediate interactions between cancer cells and platelets, leukocytes, and endothelial cells, thus facilitating tumor cell invasion and metastasis. Galectins, a family of soluble proteins that bind β-galactoside-containing glycans, have been implicated in diverse events associated with cancer biology such as apoptosis, homotypic cell aggregation, angiogenesis, cell migration, and tumor-immune escape. Consequently, individual members of these lectin families have become promising targets for the design of novel anticancer therapies. During the past decade, a number of inhibitors of lectin–glycan interactions have been developed including small-molecule inhibitors, multivalent saccharide ligands, and more recently peptides and peptidomimetics have offered alternatives for tackling tumor progression. In this article, we review the current status of the discovery and development of chemical lectin inhibitors and discuss novel strategies to limit cancer progression by targeting lectin–glycan interactions.

## Introduction: Deciphering the “Glyco-Code” in Cancer

Cancer is a leading cause of death worldwide and represents one of the biggest challenges faced by medicine. Novel biological therapies such as tumor-antigen targeted vaccines (1, 2) and immune checkpoint blockade [i.e., monoclonal antibody (mAb)-based therapies targeting cytotoxic T-lymphocyte antigen 4 (CTLA-4) or programed cell death protein-1 (PD-1) (3–5)] have been designed to target specific determinants expressed by different tumor types and their associated stroma and immune compartments. However, due to the complexity of the tumor microenvironment (TME) and the intrinsic or acquired resistance mechanisms, only certain types of cancers can be effectively treated by these therapies (3–5). Furthermore, variable responses in patients with a similar malignancy reflect inherent heterogeneity among different tumor types (5). As technology advances, genomics studies have unveiled specific genetic signatures, which enabled tailored treatments and personalized cancer therapy to move a step closer to fruition. In time, other high-throughput technologies have emerged to expand personalized medicine beyond genomics, including proteomics and more recently, glycomics (6).

Glycosylation is the most abundant posttranslational modification: all cell surface and secreted glycoproteins must travel through the endoplasmic reticulum and the Golgi compartments, where addition of carbohydrates take place. The structure and nature of glycans strongly influence various functional aspects of glycoproteins such as cellular localization, turnover, protein quality control, and receptor–ligand interactions. The structural diversity of glycans, a key aspect that governs their role as information carriers, results from the concerted action of a number of glycosyltransferases and/or glycosylhydrolases that build and remodel their structure, generating a variety of glycoforms for a specific peptide sequence and allowing both cell-type and protein-specific glycan expression patterns (7, 8). Taking into consideration the ubiquitous presence of glycoconjugates on the cell surface, the fact that certain human diseases (including cancer) display altered glycan processing pathways is not surprising to glycobiologists (9, 10).

During the last decades, and as a result of advances in glycomics and glycoproteomics technologies, aberrant cell surface glycosylation has been considered an important hallmark of cellular oncogenesis and tumor progression. Simultaneous alterations of the overall glycome were identified in several types of cancer, where a differential glycan profile could be found not only in tumor cells themselves and the associated microenvironment (stromal fibroblasts, endothelial cells, and immune infiltrating cells) but also in serum glycoproteins (i.e., acute phase proteins), revealing potential glycobiomarkers of malignancy (11–13). It is now well established that aberrant glycosylation can promote tumor cell invasion and metastasis, as these processes involve cell detachment, intravasation, transport, attachment, extravasation, and angiogenesis (14). A long-standing and still unresolved question is whether aberrant glycosylation is a cause or a consequence of tumorigenesis.

**Figure 1:**
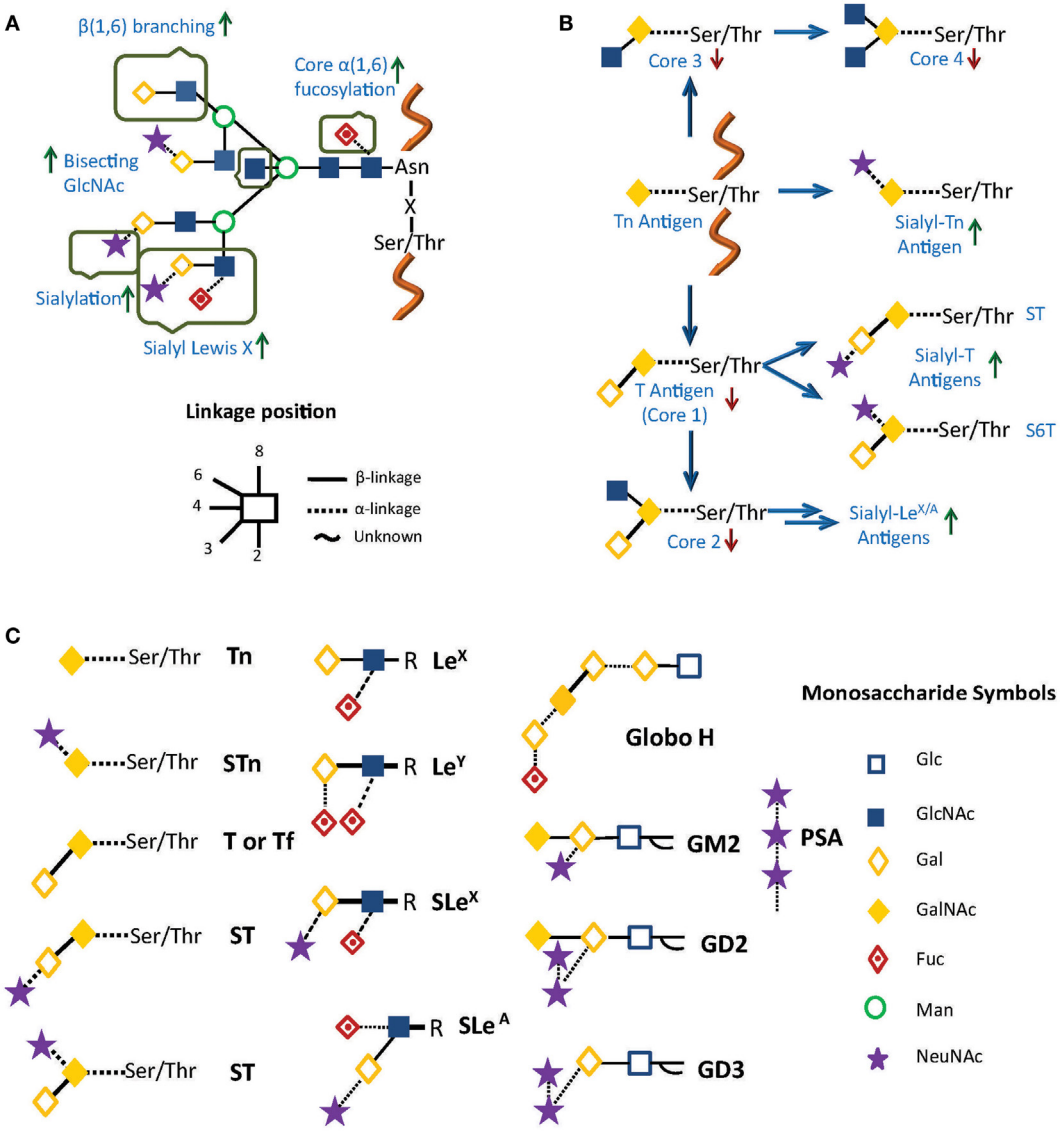
**Common alterations observed in cancer in (A) *N*-glycosylation and (B) *O*-glycosylation**. Green and red arrows represent increased and decreased expression of glycan structures, respectively. **(C)** Principal tumor-associated carbohydrate antigens (TACAs). In all cases, glycans are represented using a combination of the Oxford (21) and the Consortium for Functional Glycomics formats (http://www.functionalglycomics.org/).

Aberrant glycosylation in cancer is usually associated with poor prognosis, and may be present in different glycoconjugates, not only in *N*- and *O*-glycans on cell surface glycoproteins (15) but also in glycolipids and glycosaminoglycans (GAGs) (13, 16). These altered structures constitute the so-called tumor-associated cancer antigens (TACAs; Figure 1), and implicate not only the under- or overexpression of naturally occurring glycans but also the neo-expression of others. Glycan alterations vary depending on the type of cancer, but for *N*-glycans, they can include differential expression of blood group Lewis-related antigens such as Lewis X (Le^X^), Lewis Y (Le^Y^), sialyl Lewis X (SLe^X^), and sialyl Lewis A (SLe^A^), increased synthesis of polylactosamine chains, increased β(1 → 6) branching of *N*-linked glycans, core α(1 → 6)-fucosylation, outer arm α(1 → 2)- and α(1 → 3)-fucosylation, and changes in sialylation, among others (11, 17, 18) (Figure 1A). For *O*-glycans and glycolipids, expression of TACA include mucin-related (*O*-linked) GalNAc (Tn), sialyl Tn (STn), Thomsen–Friedenreich antigen (Tf), polysialic acid (PSA), glycosphingolipid Globo-H, and gangliosides GM2 and GD2/GD3 (2, 9, 14, 19, 20) (Figures 1B,C).

It has been clearly demonstrated that these changes in glycosylation are dependent on biochemical factors such as availability of nucleotide sugar pools (activated donors for glycan biosynthesis), and differential expression of certain glycosyltransferases (17). As examples of important glycosylatransferases involved in *O*-glycan aberrant biosynthesis, ppGalNAc6 (GALNT6, one of the UDP-*N*-acetyl-d-galactosamine: polypeptide *N*-acetylgalactosaminyltransferases responsible for the initiation of mucin-type *O*-glycosylation) is upregulated in breast cancer (22) and has been also postulated as a potential marker associated with venous invasion in gastric carcinoma (23). On the other hand, GALNT9, another member of this family, has been described as a prognostic marker in neuroblastoma (24). In prostate cancer, overexpression of GCNT1 [β(1 → 6)-*N*-acetylglucosaminyltransferase, involved in core 2 *O*-glycan biosynthesis] was associated with higher levels of core 2 *O*-SLe^x^ in prostate specific antigen (PSA) (25). Regarding *N*-glycans, bisecting GlcNAc has been identified as a hallmark of epithelial ovarian cancer and mannosyl β(1 → 4)-glycoprotein β(1 → 4)-*N*-acetylglucosaminyltransferase (MGAT3), the glycosyltransferase involved in its biosynthesis, showed a clear upregulation in ovarian cancer (26). Finally, Wang et al. described an upregulation of Fut8, a fucosyltransferase that decorates the *N*-glycan core, in hepatocellular carcinoma (HCC), and core fucosylation was proposed as a prognostic marker as well as a therapeutic target for HCC (27). The role of *N*-glycans and *O*-glycans in cancer has been thoroughly reviewed in previous publications (17, 28, 29).

Even though there is abundant evidence on the important role of altered expression of glycosyltransferases in tumor cells, the regulation of glycan-related pathways is still far from clear. In the last few years, studies describing the influence of DNA methylation (26, 30) as well as cytokine levels (31) have shed light on these issues. Finally, a recent article describing the mutational landscape of aberrant glycosylation in colon cancer has shown specific mutations associated to glycosyltransferases in patients, particularly in B3GNT2, B4GALT2, and ST6GALNAc2. These significant findings indicate that functionally deleterious mutations in glycosyltransferase genes in part underlie aberrant glycosylation and contribute to the pathogenesis of molecular subsets of colon and other gastrointestinal malignancies (32).

**Figure 2:**
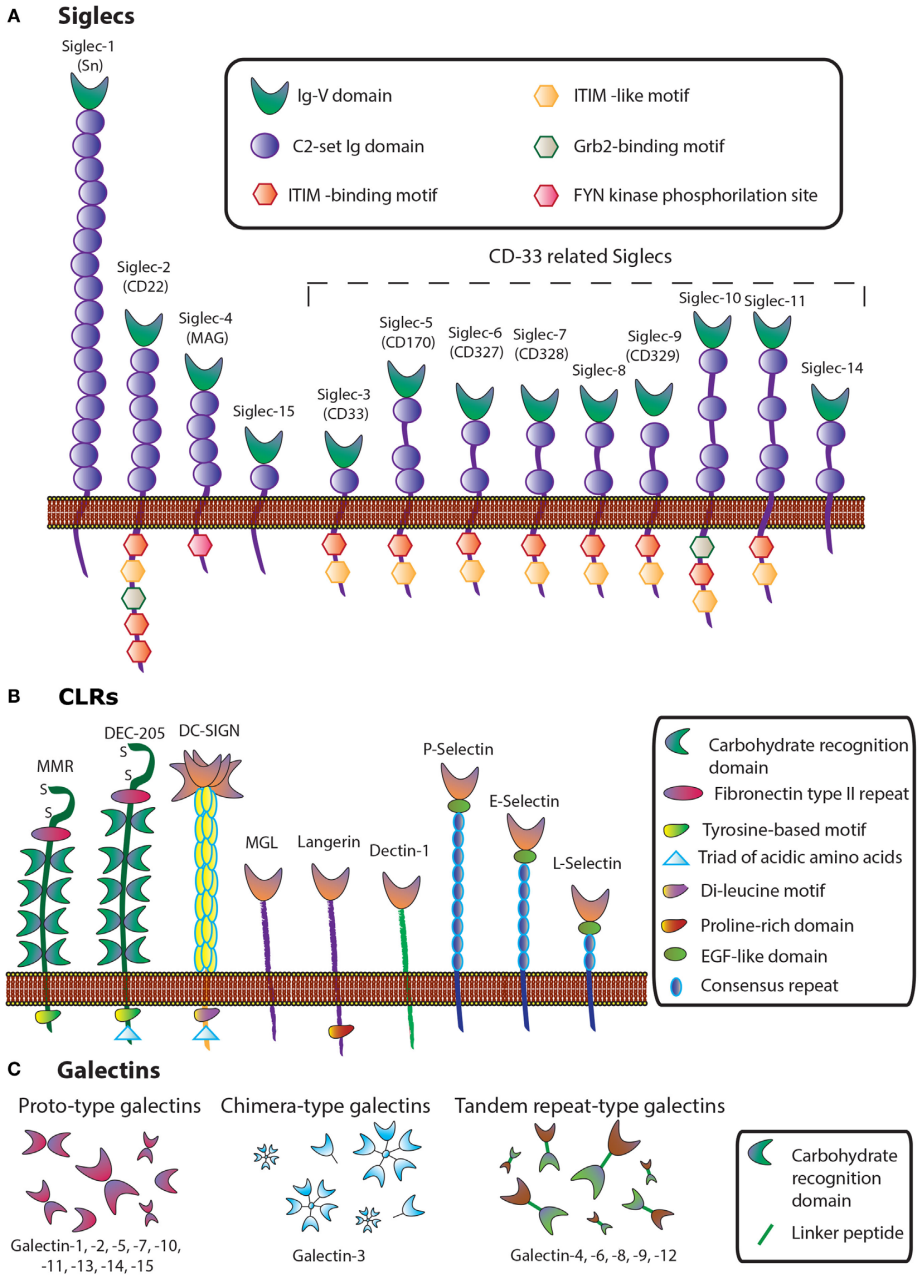
**Schematic representation of three lectin families: (A) siglecs, (B) C-type lectins, and (C) galectins**.

These glycosylation changes, complex as they are, trigger different biological processes *via* interaction with an evolutionarily divergent family of glycan-binding proteins or lectins. Lessons learned from knockout and transgenic models in physiologic and pathologic settings revealed major roles for lectin–glycan interactions in immune cell homeostasis, controlling regulatory cell programs, and activating tolerogenic circuits that orchestrate tumor-immune escape mechanisms (33, 34). In this review, we focus on therapeutic strategies, based on chemical inhibition of three different lectin families, namely sialic acid-binding immunoglobulin (Ig)-like lectins (siglecs), C-type lectin receptors (CLRs), and galectins, which play relevant roles in cancer (Figure 2).

### Siglecs and Immune Evasion in Cancer

Siglecs, also known as the I-type lectin family, constitute a family of sialic acid binding Ig domain-containing lectins that are mainly found on cells of the immune and hematopoietic system (35) (Figure 2). From a structural viewpoint, siglecs are transmembrane type I receptors bearing 2–16 extracellular C2-set Ig domains, with an extracellular N-terminal V-set Ig (Ig-V) domain responsible for the binding of sialoside ligands (36), a single transmembrane domain, and varying lengths of cytosolic tails (37) (Figure 2A). Siglecs are typically classified into two functionally diverse subsets. The most distantly interrelated group (25–30% sequence identity) includes Siglec-1 (Sialoadhesin, Sn), -2 (CD22), -4 [myelin-associated glycoprotein (MAG)], and -15. The second group represents the rapidly evolving CD33-related Siglecs, which have high homology to CD33 in their extracellular domains (50–85% identity) and comprises Siglec-3 (CD33), -5, -6, -7, -8, -9, -10, -11, and -14 (35, 37, 38).

Siglecs are primarily expressed in B cells, macrophages, dendritic cells (DCs), and eosinophils and have been implicated in both innate and adaptive immunity. They play important roles in host–pathogen interactions, cell–cell communication, and regulation of immune tolerance (39), maintaining immune homeostasis and regulating inflammatory processes (37). With respect to innate immunity, Siglecs have been involved in pathogen internalization and immune evasion, attenuation of damage-associated molecular pattern (DAMP)-mediated inflammation, and inhibition of natural killer (NK) cell function. In adaptive immunity, they act as modulators of T-cell activation and polarization as well as regulators of B cells and plasmacytoid DCs (38).

**Figure 3:**
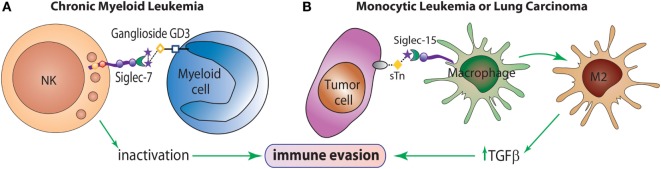
**The role of siglecs in immune evasion mechanisms**. **(A)** Siglec-7 is expressed predominantly on NK cells and inhibits NK cell cytotoxicity toward target cells overexpressing the α(2 → 8)-disialic acid-bearing ganglioside, GD3. **(B)** Siglec-15 recognizes the tumor sialyl-Tn (sTn) antigen and transduces an intracellular signal leading to enhance TGF-β secretion and polarization toward an M2-like macrophage profile, which contributes to tumor progression.

Many siglecs have been studied as potential targets for the design of therapeutic agents for the treatment of inflammatory, autoimmune, allergic, and infectious diseases (35). Even though changes in sialylation may modulate tumor cell invasion or metastasis, the involvement of siglecs in tumor immunity is currently being explored. For example, Siglec-2 (CD22) has been implicated in B-cell activation in non-Hodgkin Lymphoma (40), and Siglec-7 has been shown to exert a pivotal role in tumor escape by inactivation of NK cells (41) (Figure 3A). Siglec-3 (CD33) is expressed on malignant blast cells in 85–90% of Acute Myeloid Leukemia cases, while is absent on normal hematopoietic pluripotent stem cells (42). Takamiya et al. reported that Siglec-15, which preferentially recognizes sialyl-Tn antigen (Figure 1), induced a M2-like immunosuppressive macrophage phenotype and upregulated TGF-β secretion in human monocytic leukemia cells and human lung carcinoma cells (43) (Figure 3B). Furthermore, interactions between Siglec-4a (MAG) and the mucin MUC1 enhanced adhesion of pancreatic cells and stimulated pancreatic cancer cell perineural invasion (44). Other siglecs have been correlated with tumor progression, such as Siglec-9, involved in tumor-immune evasion, and Siglec-12, which was found to be overexpressed on human prostate epithelial carcinomas (45).

### Role of Selectins in Metastasis and Tumor-Associated Inflammation

C-type lectins comprise a diverse family of calcium-dependent glycan-binding proteins that play essential immunological roles as adhesion and signaling receptors in inflammation, tumor progression, and viral infections (46). This lectin family is classified into 17 different subgroups depending on their C-type lectin domains and their structures. Some representative subsets are collectins, endocytic receptors [Mannose Receptor (MR), Dec-205], DC receptors [dendritic-cell specific intracellular adhesion molecule 3-grabbing non-integrin (DC-SIGN)], macrophage galactose-binding lectin (MGL), Langerin, and selectins (L-Selectin, P-Selectin, and E-Selectin) (Figure 2B).

**Figure 4:**
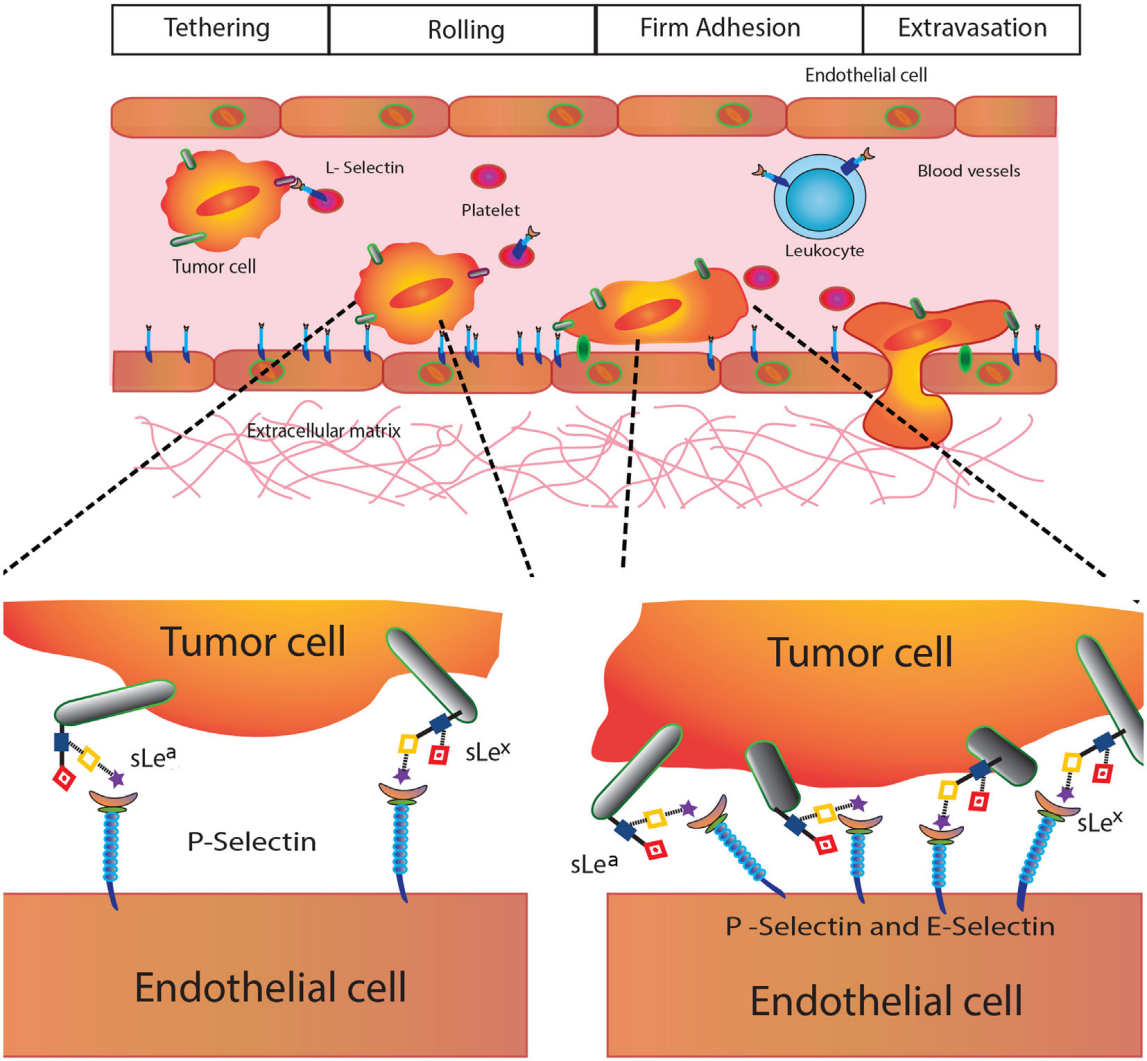
**Selectins in cancer biology**. Selectins play different roles in tumor biology including modulation of platelet–cancer cell interactions (P-selectin), promotion of tumor cell adhesiveness, extravasation and metastasis (E-selectin), and leukocyte trafficking and hematogenous metastasis (L-selectin).

Given their biological and clinical relevance, we will focus here on Selectins, C-type transmembrane lectins that mediate leukocyte trafficking and specific adhesive interactions of leukocytes, platelets, and endothelial cells with tumor cells (47). These lectins are present on endothelial cells (E-Selectin), leukocytes (L-Selectin), and platelets (P-Selectin) (46), and preferentially bind glycans containing SLe^X^ and SLe^A^ glycoepitopes (Figure 1), which are abundantly expressed in several tumor types. In the TME, selectins are functionally relevant in the context of leukocyte recruitment, tumor-promoting inflammation, and acquisition of metastatic potential (36) (Figure 4).

P-Selectin (CD62P) is involved in tumor growth and metastasis, as it mediates interactions between activated platelets and cancer cells contributing to tumorigenesis (47). E-Selectin (CD62E) also play major roles in cancer cell adhesiveness at different events of the metastatic cascade, promoting tumor cell extravasation (48). Finally, L-Selectin (CD62L), constitutively expressed on leukocytes, regulates tumor–leukocyte interactions and promotes cell adhesion and hematogenous metastasis by favoring emboli formation (49).

Because of their critical involvement in cancer metastasis, several research groups have developed therapeutic strategies based on disruption of selectin–glycan interactions with the ultimate goal of controlling inflammation and metastasis (48).

### The Galectin–Glycan Axis in Cancer Development

Galectins, a family of highly conserved glycan-binding soluble lectins, are defined by a conserved carbohydrate recognition domain (CRD) and a common structural fold (50). Based on structural features, mammalian galectins have been classified into three types: prototype galectins (Gal-1, -2, -5, -7, -10, -11, -13, -14, and -15, containing one CRD and existing as monomers or dimerizing through non-covalent interactions), tandem repeat-type galectins (Gal-4, -6, -8, -9, and -12), which exist as bivalent galectins containing two different CRDs connected by a linker peptide, and finally, Gal-3, the only chimera-type member of the galectin family (Figure 2). Their distribution in mammalian tissues is diverse. While Gal-1 and -3 are detected ubiquitously, other galectins are more specifically located, such as Gal-2 and -4, which are preferentially found in the gastrointestinal tract (51, 52), Gal-7 is highly abundant in the skin (53), Gal-10 in eosinophils (54), and Gal-12 in adipose tissue (55, 56).

The ability of galectins to modulate different events in tumorigenesis and metastasis makes them attractive targets for cancer therapy (57, 58), controlling malignant transformation (59), apoptosis (60), cell-cycle progression (61), angiogenesis (62, 63), tumor metastasis (64, 65), and tumor immune escape (66). Galectins contribute to immune tolerance and escape through apoptosis of effector T cells (67), regulation of clonal expansion, function of regulatory T cells (Tregs) (64), and control of cytokine secretion (68). Expression levels for some galectins also change during malignant transformation, confirming their essential roles in cancer progression (69). Among the galectin family members, in this review, we will focus on Gal-1 and Gal-3, the two most extensively studied galectins, which have key roles during cancer progression.

**Figure 5:**
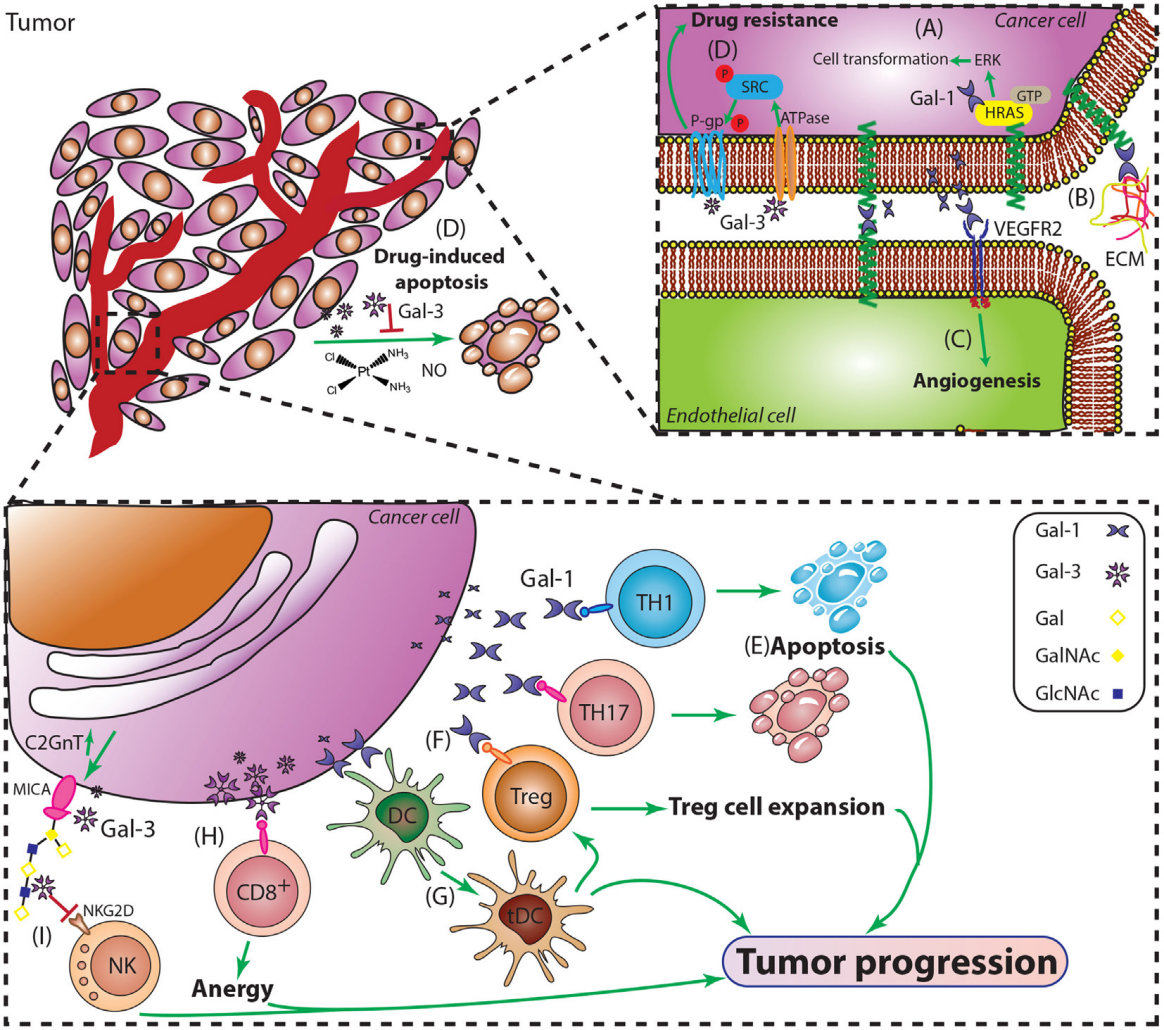
**Major roles of Gal-1 and Gal-3 during cancer progression**. **(A)** Intracellular Gal-1 triggers activation of the H-Ras/ERK cascade leading to malignant transformation. **(B)** Gal-1 promotes adhesion of tumor cells to extracellular matrix and endothelial cells, critical steps during early stages of tumor invasion and metastasis. **(C)** Gal-1 induces tumor angiogenesis by engaging key proangiogenic pathways including VEGF-like signaling. **(D)** Resistance to apoptosis is essential for cancer cell survival and plays a role in tumor progression. Gal-3 suppresses apoptosis induced by cisplatin, nitric oxide (NO), or radiation, through interactions with P-gp and Na^+^/K^+^-ATPase, thus promoting tumor cell survival. **(E)** Gal-1 selectively deletes T_H_1 and T_H_17 cells and **(G)** promotes the differentiation of tolerogenic DCs (tDCs). **(F)** Gal-1 promotes expansion of CD4^+^CD25^+^Foxp3^+^ regulatory T cells (Tregs) and amplifies their immunosuppressive activity. **(H)** Gal-3 induces anergy of CD8^+^ T cells by distancing the TCR from CD8 molecules. **(I)** Gal-3 impairs NK cell function by inhibiting the interactions between the heavily *O*-glycosylated tumor-derived MICA and the NK cell activating receptor NKG2D.

Gal-1, abundantly secreted by almost all malignant tumor cells, has been characterized as a major promoter of an immunosuppressive protumorigenic microenvironment (67). This lectin induces selective apoptosis of T_H_1 and T_H_17 effector T cells, without affecting T_H_2 cells due to differential sialylation of cell surface glycoproteins (67, 70) (Figure 5). In recent years, the immunosuppressive activity of Gal-1 has also been extended to differentiation and expansion of CD4^+^CD25^+^Foxp3^+^ Tregs (64) and, similarly to other galectins, controls cell surface retention and signaling thresholds of a number of glycosylated receptors. However, its immunoregulatory activity is not limited to T cell populations: Gal-1 also promotes differentiation of tolerogenic dendritic cells (tDCs) (71) and controls tissue emigration of immunogenic, but not tDCs (72). The tolerogenic effects induced by this lectin have been substantiated by the work of Kuo and colleagues, who found that Gal-1-induced tDCs in lung cancer also favored the induction of Tregs (73). Furthermore, in addition to its immune inhibitory effects, Gal-1 can also favor tumor development and progression through promotion of tumor angiogenesis, favoring vascular endothelial growth factor (VEGF) signaling, and promoting endothelial cell proliferation, adhesion, migration, and resistance to apoptosis (34, 74). Finally, Gal-1 has been reported to contribute to heterotypic adhesion of tumor cells to extracellular matrix and endothelial cells, critical steps during the early stages of tumor invasion and metastasis (74, 75). Finally, within the intracellular compartment, Gal-1 also interacts with oncogenic H-Ras–guanosine triphosphate (H-Ras–GTP) through its farnesyl group, enhancing H-Ras-mediated cell transformation through ERK1/2 signaling (76) (Figure 5).

Gal-3, another member of the family, has shown prominent protumorigenic effects in a multiplicity of tumors. This multifunctional protein has demonstrated different effects depending on its subcellular localization (77) (Figure 5). A pioneer work by Raz et al. reported a correlation between higher Gal-3 expression and increase incidence of experimental lung metastases in mouse models (78). Since then, compelling evidence has implicated Gal-3 expression with different aspects of cancer biology including cell adhesion, migration, angiogenesis, and immune escape (79, 80).

Thomsen–Friedenreich glycoantigen (Tf; Figure 1) is expressed in up to 90% of human carcinomas (81) and is a major cancer cell surface carbohydrate ligand for Gal-3. Similarly to Gal-1, Gal-3 signaling contributes to tilt the balance toward immunosuppressive TMEs by interacting with specific glycans, and impairing antitumor responses. In this regard, Gal-3 has been shown to promote anergy of tumor infiltrating lymphocytes (TILs) (82). Furthermore, Tsuboi et al. described a new mechanism of tumor escape involving Gal-3 and NK cells in bladder cancer (83); the authors demonstrated that overexpression of core 2 β(1→6)-*N*-acetylglucosaminyl transferase 1 (C2GnT1), a glycosyltransferase responsible of generating branched core-2 *O*-glycans that can be elongated with poly-*N*-acetyllactosamine (LacNAc) sequences, negatively controls the activity of tumor-associated major histocompatibility complex class I-related chain A (MICA). Interactions between polyLacNAc and Gal-3 reduced the affinity of MICA to the NK cell receptor NKG2D, thereby impairing NK cell activation and their antitumor activity [(71) Figure 5]. However, Gal-3 not only assists tumor escape by inhibiting immune responses; it also promotes tumor cell survival by hampering drug-induced apoptosis by cisplatin, nitric oxide (NO) or radiation, through phosphorylation, translocation, and regulation of survival signaling pathways (84, 85) (Figure 5). The mechanisms underlying Gal-3 protection of drug-induced apoptosis has recently been reported by Harazono et al. (86, 87), who showed that interaction of this lectin with Na^+^/K^+^-ATPase activated SRC tyrosine kinase, subsequently inducing phosphorylation of P-glycoprotein (P-gp) and enhancing its ATPase activity. These effects contribute to decrease sensitivity to doxorubicin-mediated cell death (72, 73).

## Structure, Function, and Clinical Prospects of Lectin-Inhibitory Antitumor Chemical Agents

### Antitumor and Antimetastastic Agents Targeting Selectins

A variety of approaches have been designed to tackle selectin-mediated inflammation and metastasis through chemical inhibition. As a result, sulfated polysaccharides, modified glycans, and glycopeptides have been postulated as pharmacological selectin inhibitors (Table 1).

**Table 1:** **Structure and clinical applications of different anti-selectin agents described in this review**.

Type	Name	N°	Structure	Tumor type tested	Reference
Heparin derivatives	Heparin sulfate	*1*	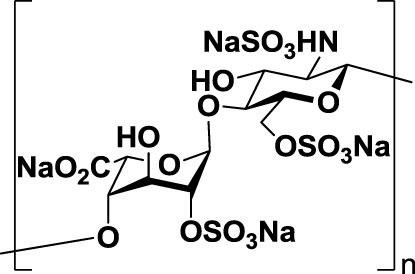	Human sarcoma, melanoma, and colon carcinoma	(88)
	Dermatan sulfate	*2*	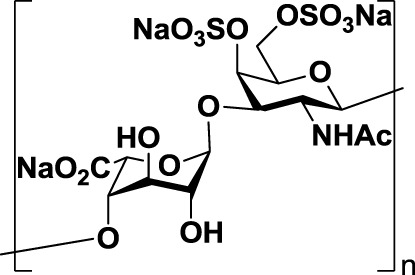	Colon carcinoma and melanoma	(89)
	6-*O*-sulfated chitosan	*3*	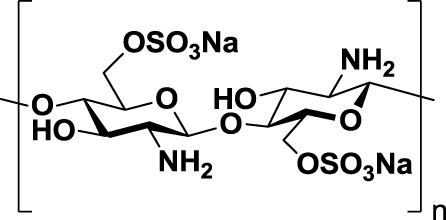	Human melanoma	(90)
SLe^X^ glyco-mimetics	Gal-α-(1 → 1)-β-Man C-fluorinated analogs	*4*	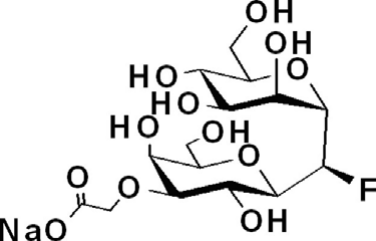		(91)
	NMSO3	*5*	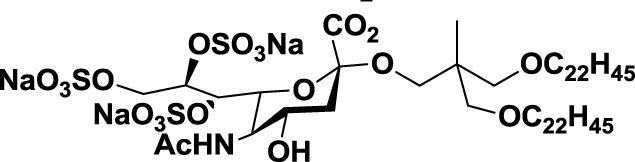	Human promyelocytic leukemia	(92)
Glyco-peptides	Fluorinated C-manno-peptides	*6*	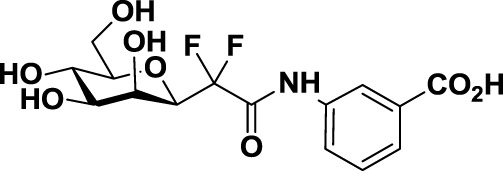		(93)
	SLe^X^-glyco-peptide	*7*	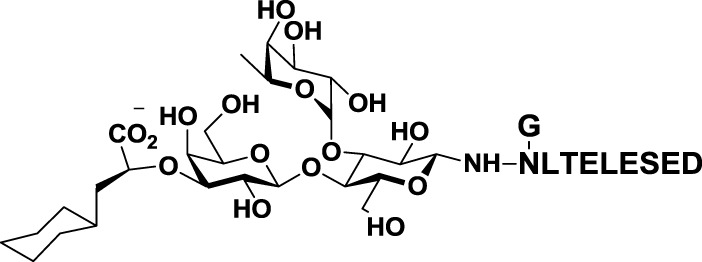		(94)

#### Sulfated Oligosaccharides

Heparin (*1*, Table 1), a highly sulfated polysaccharide from the GAG family, is composed of a repetitive disaccharide unit containing glucosamine and glucuronic/iduronic acid residues with a high degree of sulfation (95). Heparins and its derivatives, traditionally used as anticoagulants, have recently emerged as attractive compounds for selectin inhibition showing promising antimetastatic activity through disruption of P- and L-selectin-mediated adhesion of tumor cells (96). In an attempt to reduce the anticoagulant activity of heparin and enhance its affinity toward selectins, different chemical modifications such as *N*-acetylation, succinylation, *O*-desulfation, and reduction have been performed (97, 98). Heparins of different molecular weight have been successfully tested in tumor models such as melanoma, sarcoma, breast, and colon adenocarcinoma demonstrating inhibitory effects on tumor metastasis. However, they had limited effects on tumor growth, showing that heparin mediates interactions of tumor cells with host cells and the extracellular matrix during the metastatic process (99). Heparin and its derivatives have been tested in numerous clinical trials, but mostly as anticoagulant agents for prevention of cancer-related thrombosis (100).

Other negatively charged polysaccharides have also been evaluated as selectin inhibitors. Dermatan sulfate (*2*, Table 1), a sulfated polysaccharide bearing a [→4)-IdoA(2S)-β(1 → 3)-GalNAc-β-(1→] repetitive unit, has shown to be an effective P-selectin inhibitor *in vitro* for colon carcinoma and melanoma in experimental models (89). 6-*O*-Sulfated chitosan (*3*, Table 1), a modified linear β-(1 → 4)-glucosamine polysaccharide, has also demonstrated the ability to reduce interactions of P-selectin with melanoma cells *in vitro* (90).

Taken together, these findings suggest that inhibition of selectin–glycan interactions by charged polysaccharidic agents could become an alternative therapy for inhibition of tumor metastasis. However, further analyses are necessary not only to unravel the mechanisms underlying the pharmacological activity of these compounds but also to reduce the risk of heparin-induced anticoagulant-related side effects.

#### Glycomimetics

As selectins preferentially bind to sulfated or fucosylated oligosaccharides such as SLe^X^ or SLe^A^ (Figure 1), Lewis-type glycomimetic analogs emerged as interesting decoys for disruption of selectin-mediated processes. Non-reductive disaccharide Gal-α-(1 → 1)-β-Man became a promising building block for SLe^X^ glycomimetics, and a number of research groups have explored fluorinated C-glycosyl analogs (*4*, Table 1) in an attempt to improve the pharmacokinetics of these ligands (91, 101). On a different approach, Shodai et al. designed NMSO3 (*5*, Table 1), a sulfated derivative of sialic acid, which demonstrated to be a good inhibitor of P-selectin-mediated tumor cell adhesion (92).

#### Glycopeptides

Besides carbohydrates, glycopeptides were also evaluated as selectin blockers. Flexible difluorinated C-mannopeptides (*6*, Table 1) were synthesized and tested as E- and P-selectin inhibitors, exhibiting moderate binding affinities (93). Starting from SLe^X^, Filser et al. designed high-affinity synthetic glycopeptides for E-selectin inhibition, bearing a peptide sequence from the natural ligand E-selectin ligand-1 (ESL-1) and replacing the sialic acid with a cyclohexyl moiety (***7***, Table 1). These compounds confirmed that the peptide moiety was essential for selectin binding and exhibited encouraging IC_50_ values in the low micromolar range (94). However, no experiments have yet corroborated their efficacy *in vitro* or *in vivo*.

### Siglec Inhibition in Cancer Treatment: Sialic Acid Derivatives

**Figure 6:**
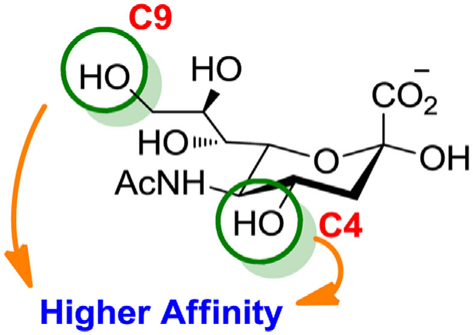
**Modifications of neuraminic acid leading to high-affinity siglec blockers**. Green circles denote the main positions modified in search of high-binding affinity and selective siglec inhibitors.

Compared to the advances made for selectins or galectins, the role of siglecs during cancer progression has not been studied in such detail. Thus, there have been fewer reports on siglec inhibitors as anticancer agents. Promising approaches reported in literature include C4- and C9-modified sialic acid derivatives (Figure 6). Siglec-7, which belongs to the CD33-related siglec type, preferentially binds internally branched α(2 → 6) sialic acid and is primarily expressed on NK cells. This lectin has been reported to favorably interact with melanoma or neuroblastoma cancer cells that overexpress GD3, an α(2 → 8) disialic acid-bearing ganglioside, thus inhibiting NK cell cytotoxicity as an immune evasion mechanism (102). In an attempt to disrupt Siglec-7–GD3 interactions as a potential cancer therapeutic strategy, Attrill et al. described the design of sialic acid derivatives as inhibitors of Siglec-7 signaling (103). One of these ligands, oxamido-Neu5Ac [*8*, Table 2, methyl α-9-(amino-oxalyl-amino)-9-deoxy-Neu5Ac] exhibited a twofold decrease in the IC_50_ value (1.6 mM) for inhibition of Siglec-7 *in vitro*, compared to the canonical ligand methyl-α-Neu5Ac (>3.0 mM) (103).

**Table 2:** **Anti-siglec agents described in this review**.

Name	N°	Structure	Tumor type tested	Reference
Oxamido-Neu5Ac	*8*	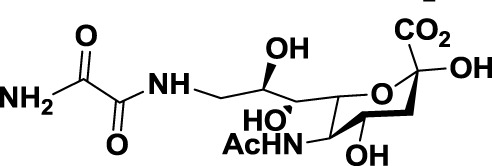		(103)
BPC-Neu5Ac	*9*	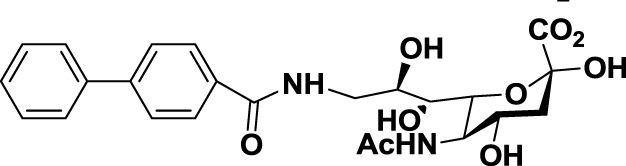	Non-Hodgkin’s lymphoma	(104)
BPC-Neu5Ac-Dox liposome	*10*	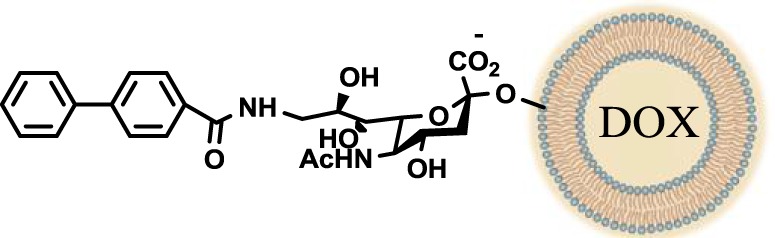	Non-Hodgkin’s lymphoma	(105)
9-BPC-4-mNPC-Neu5Ac	*11*	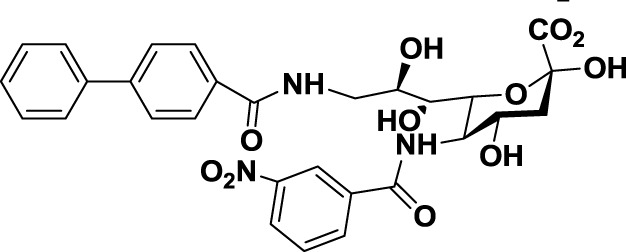		(106)

Another interesting approach was reported by Kelm et al. who described the synthesis of sialic acid derivatives as high-affinity inhibitors of Siglec-2 (106). CD22 or Siglec-2 is an antigen widely expressed on normal and malignant B cells and plays a primary role in B-cell activation. Thus, it has become an interesting target for the treatment of autoimmune diseases and B-cell derived non-Hodgkin’s Lymphoma (40). In 2002, Kelm et al. reported the synthesis of a C9-modified Neu5Ac, namely methyl-α-9-*N*-(biphenyl-4-carbonyl)-amino-9-deoxy-Neu5Ac (BPC-Neu5Ac, *9*, Table 2), which exhibited a >200-fold relative inhibitory potency (rIP) for human CD22 than Me-α-Neu5Ac (104). More recently, Paulson and colleagues used this potent Siglec-2 inhibitor for the design of doxorubicin-loaded liposomal nanoparticles bearing BPC-Neu5Ac (*9*, Table 2) ligands to target B cell lymphoma (105). The CD22-targeted BPC-Neu5Ac-Dox liposomes (*10*, Table 2) provided a significant increase in survival rates when injected into a xenograft model of human B-cell lymphoma. Interestingly, this delivery strategy exhibited cytotoxic effects toward malignant B cells in patients with hairy cell leukemia, marginal zone lymphoma, and chronic lymphocytic leukemia. In 2013, Kelm et al. presented a further optimized novel glycan inhibitor with modifications on both Neu5Ac C4 and C9 positions (106). This compound, methyl 9-biphenylcarboxamido-4-*m*-nitrophenylcarboxamido-4,9-dideoxy Neu5Ac (9-BPC-4-mNPC-Neu5Ac, *11*, Table 2) presented a 14-fold decrease in the IC_50_ value toward Siglec-2, thus emerging as a promising lead agent, although its activity has not yet been tested *in vitro* or *in vivo*.

### Therapeutic Strategies Targeting Galectin–Glycan Interactions in the TME

Given the key protumorigenic, prometastatic, and immunosuppressive activities of galectins and their roles in tumor resistance to antiangiogenic therapies, important efforts have been made in the design of high-binding affinity specific inhibitors. Here, we describe a number of successful strategies to chemically disrupt galectin–ligand interactions and discuss advantages, limitations, and obstacles in their translation to clinical settings.

#### Low-Molecular Weight Inhibitors

##### A First Step: β-Galactoside Ligands

Since galectins share the structurally conserved CRD that defines their natural ligands, the initial approach was the design of β-galactoside inhibitors targeting the carbohydrate-binding site (107). Specific modifications of the galactose-based structures have led to the design of good inhibitors with dissociation constants in the micromolar range (20–300 μM) (108, 109). However, the diverse chemical modifications tested on this monosaccharide did not achieve enough improvement on galectin affinity, and in consequence, galactose-based monosaccharide inhibitors have not been tested in cultured cells or *in vivo*.

The second approach for the design of galectin inhibitors was the use of chemically modified natural galectin ligands, such as the disaccharides lactose (Lac) or *N*-acetyllactosamine (LacNAc). Ingrassia et al. reported an unspecific lactosylated steroid (*12*, Table 3) that induced increased survival rates when administered in experimental models of mouse lymphoma and human glioblastoma (110). Furthermore, Iurisci et al. reported the use of allyl-lactoside (*13*, Table 3) as ligand of Gal-1 and Gal-3, inhibiting homotypic cell aggregation in human melanoma cells, and promoting apoptosis of small cell lung carcinoma cells (111). Although Lac and LacNAc are more promising scaffolds than galactose for the design of galectin inhibitors since both disaccharides present higher binding affinity than galactose (*K*_D_ ≈ 90–500 μM), they are both sensitive to enzymatic hydrolysis by glycosidases.

**Table 3:** **Structure and applications of different anti-galectin agents described in this review**.

Type	Name		N°	Structure	Tumor type tested	Reference
Low-molecular weight inhibitors	Lactosylated steroid		*12*	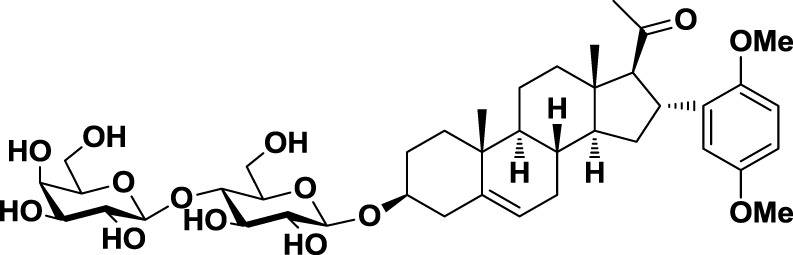	Mouse models of mouse lymphoma and human gliblastoma	(110)
	Allyl lactoside		*13*	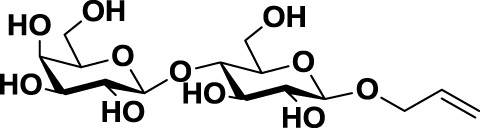	Human melanoma and small cell lung cancer cells	(111)
	Thiodigalactoside (TDG)		*14*	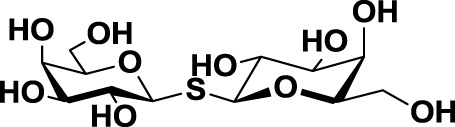	Murine lung metastasis	(112)
	3,3′-di-(3-metoxibenzamido)-thiodigalactoside		*15*	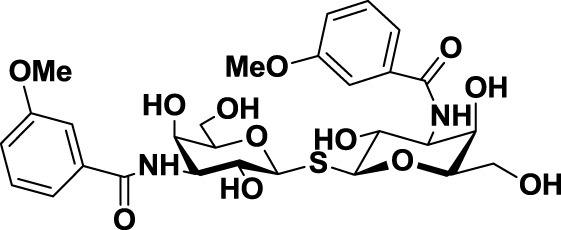		(113)
	3,3′-ditriazolyl thiodigalactoside		*16*	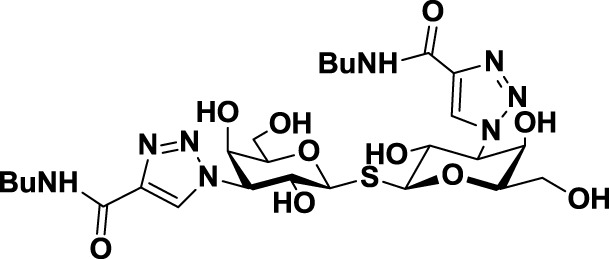		(113)
	Td131_1		*17*	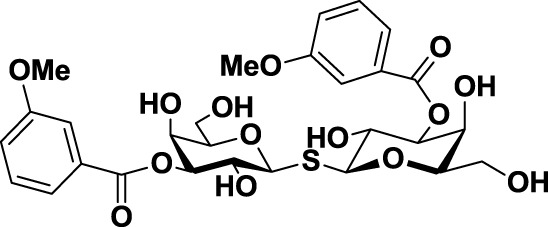	Papillary thyroid cancer	(114)
	Talosamide		*18*	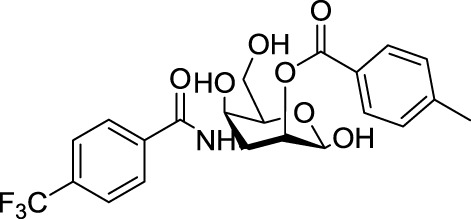		(115)
Glycoamines	Lactulose-l-leucine (Lac-l-Leu)		*19*	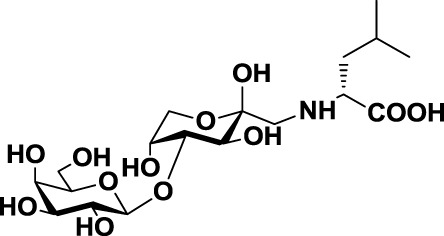	Human breast carcinoma, prostate cancer	(116–118)
	*N*-lactulose-octamethylenediamine (LDO)		*20*	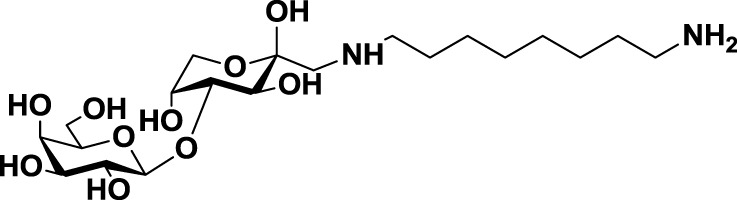	Melanoma and lung carcinoma	(119)
	*N*, *N*′-dilactulose-octamethylenediamine (D-LDO)		*21*	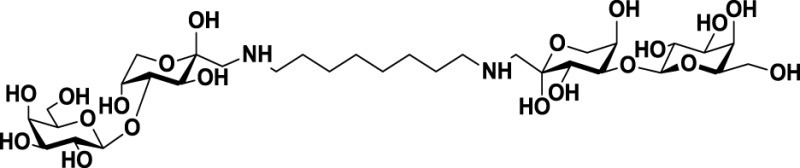		
	*N*, *N*′-dilactulose-dodecamethylenediamine (D-LDD)		*22*	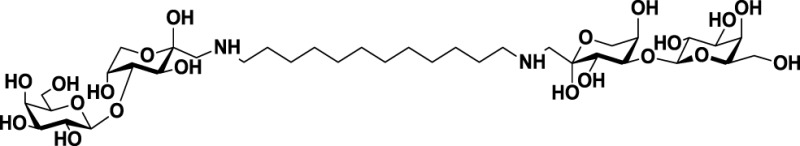		
Polysaccharide-based compounds	Pectin-derived compounds	Pectasol-C	*23*	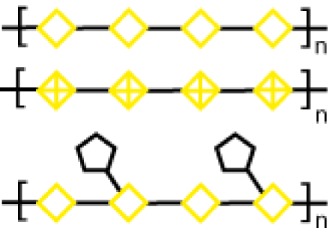	Prostate and human breast cancer	(120–124)
		GCS-100	*24*		Myeloma and leukemia	(125, 126)
		GR-MD-02	*25*	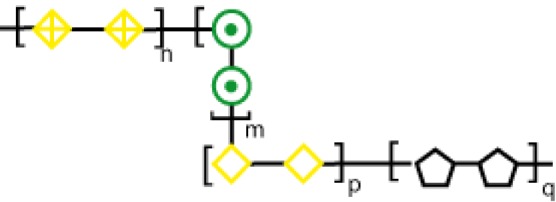	Melanoma	(127)
	Galactomannan-derived compound	GM-CT-01 (DAVANAT)	*26*	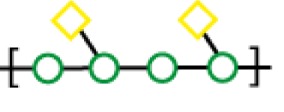	Colon cancer	(128)
Peptides	G3-A9		*27*	PQNSKIPGPTFLDPH	Breast carcinoma metastasis, prostate cancer	(129, 130)
	G3-C12		*28*	ANTPCGPYTHDCPVKR		
	Anginex		*29*	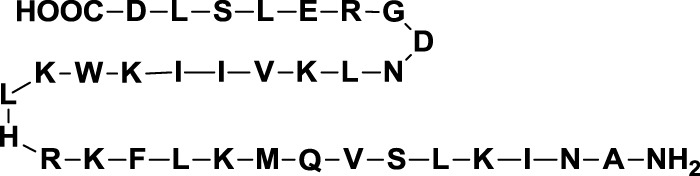	Ovarian carcinoma	(131)
Peptidomimetics	6DBF7		*30*	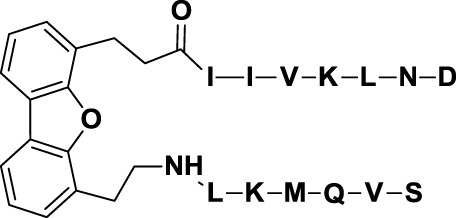	Ovarian carcinoma	(132)
	OTX008		*31*	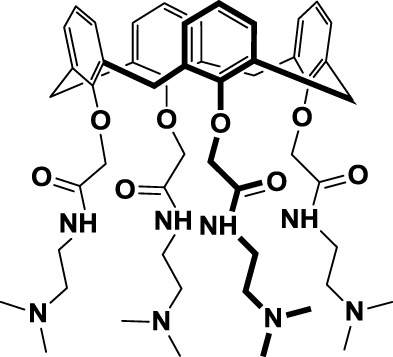	Ovarian carcinoma and murine melanoma and glioblastoma	(133, 134)

**Figure 7:**
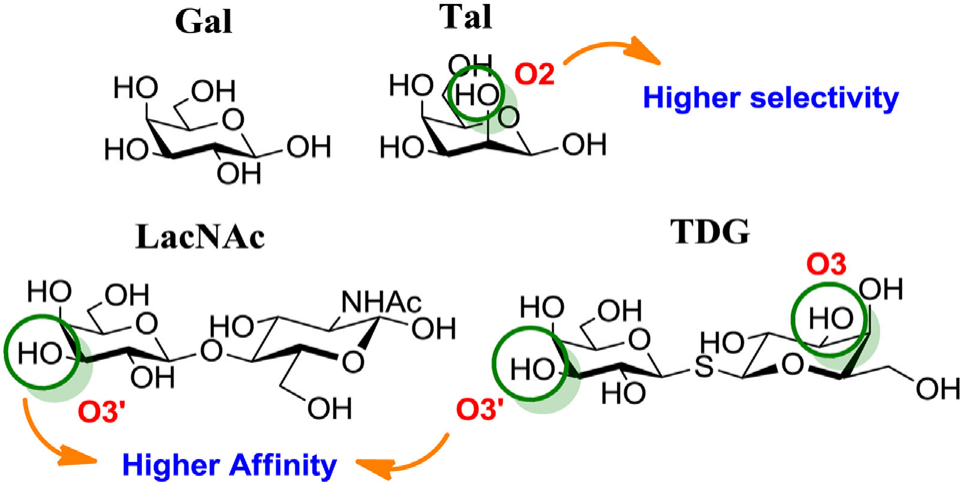
**Mono and disaccharides as building blocks to design galectin inhibitors**. Structures of galactose (Gal), talose (Tal), *N*-acetyllactosamine (LacNAc), and thiodigalactoside (TDG). Green circles denote critical modified positions in search for high-binding affinity and selective galectin inhibitors.

Chemical modifications in O2 and O3 of the galactose residue in lactose (named O2′ and O3′; Figure 7) afforded novel structures with anticancer biological activities. The Nilsson group explored galactose O3′ modifications for Gal-3 binding, showing that lactose O3′ modification with aromatic groups led to active Gal-3 inhibitors with binding affinities as low as 300–600 nM, due to additional favorable cation–π interactions with a conserved arginine residue, Arg-144, in the carbohydrate-binding site (113). However, these Gal-3 blockers showed affinities in the micromolar/low millimolar range (100–1000 μM) when tested *in vitro*.

In order to overcome the poor bioavailability of lactose derivatives, thiosugars, and in particular, thiodigalactoside (TDG; *14*, Table 3) have emerged as interesting building blocks for the design of potent galectin inhibitors, although with low selectivity (135, 136). Ito et al. demonstrated that administration of TDG significantly reduced tumor progression and metastasis *via* Gal-1 inhibition, using murine models of breast and colon adenocarcinoma (112). Moreover, intratumoral injection of TDG raised the level TILs and reduced tumor growth in models of B16 melanoma as well as 4T1 orthotopic breast cancer models (137). Symmetrical modifications of TDG at O3 and O3′ positions led to increased galectin-binding affinities, affording the most potent Gal-3 inhibitors *in vitro* reported to date (136), with *K*_D_ values as low as 29 nM (3,3′-ditriazolyl thiodigalactoside, *15*, Table 3) and 50 nM (3,3′-di-3-metoxibenzamido thiodigalactoside) (*16*, Table 3). In spite of their promising biochemical performance, *in vivo* experiments were not as encouraging. For instance Td131_1 (*17*, Table 3), an ester-modified TDG derivative, was evaluated as a Gal-3 inhibitor in papillary thyroid cancer. This compound showed concomitant increased apoptosis of cancer cells, although requiring concentrations up to 1000 times than the *K*_D_ value (114).

##### Changing Stereochemistry for Enhanced Selectivity: Talose-Based Ligands

Carbohydrates are characterized for containing multiple stereocenters, and many stereoisomers are possible including enantiomers and diastereoisomers. Talose (a C-2 epimer of galactose; Figure 7) provides an axially disposed O2 and additional protein–ligand interactions, potentially making talose-based inhibitors more selective than galactose-based ones. This approach was taken by Nilsson and collaborators, who created a new family of synthetic talosides that showed affinity and selectivity toward Gal-4 (C-terminal CRD), Gal-8 (N-terminal CRD), and Gal-3 (138). In 2011, Öberg et al. reported the synthesis of a new family of talosamides with additional modifications at C2 and C3 with aromatic moieties. The best matches for these talosamide galectin inhibitors were found against Galectin-4C (C-terminal) (115): compound *18* (Table 3) presented a *K*_D_ value of 94 μM and a good selectivity when compared to Gal-1 (1900 μM), Gal-2 (1700 μM), Gal-3 (570 μM), Gal-7 (>4000 μM), and Gal-9 (>4000 μM). These promising talose-based compounds still await detailed *in vitro* and *in vivo* evaluation.

##### Mimicking Glycans: Glycoamines on the Spot

A different approach to carbohydrate-based galectin inhibitors was proposed by Glinsky et al., who showed that a synthetic β-galactoside-containing disaccharide-amino acid conjugate, the glycoamine lactulose-l-leucine (Lac-l-Leu, *19*, Table 3), binds and inhibits Gal-3 by mimicking cancer-associated Tf antigen (Figure 1). This glycomimetic decreased the incidence of lung metastasis in human breast carcinoma xenografts (118) and also inhibited homotypic and heterotypic aggregation of prostate cancer cells *via* Gal-3 inhibition (81). Furthermore, intraperitoneal administration of Lac-l-Leu reduced bone metastasis of human prostate carcinoma cells in a mouse model (116). However, it required high concentrations (daily injections of 200 μL of Lac-l-Leu 20 μM) to disrupt Gal-3–Tf interactions.

Further studies reported the design and synthesis of a new family of lactulose amines based on *N*-lactulose-octamethylenediamine (LDO, *20*, Table 3) (119). In order to assess multivalency as potential modification to increase local concentration of these galactose-containing ligands, *N*, *N*′-dilactulose-octamethylenediamine (D-LDO, *21*, Table 3) and *N*, *N*′-dilactulose-dodecamethylenediamine (D-LDD, *22*; Table 3) were also synthesized. Compounds *10* and *11* (Table 3) exhibited interesting regulatory effects *in vitro*, such as prevention of Gal-1-mediated homotypic aggregation in melanoma cells and apoptosis of small cell lung carcinoma cells (119). In order to further develop these compounds and finally reach clinical status, additional structural studies clarifying the relevance of the aglycone moiety, and their selectivity even beyond galectins are required.

#### Does Size Matter? Polysaccharide-Derived Compounds

In addition to low-molecular weight carbohydrate-based synthetic inhibitors, natural polysaccharides have also emerged as high affinity galectin inhibitors with low toxicity for cancer treatment. Modified citrus pectin (MCP, Pectasol-C) and Davanat (GM-CT-01) are the best studied galectin blockers derived from natural sources.

##### Pectin-Based Compounds

Pectins compose a family of complex polysaccharides, which are found in high amounts in the plant primary wall. Three main pectic polysaccharides have been isolated from plant walls: homogalacturonan (HG), rhamnogalacturonan-I (RG-I), and substituted galacturonans (GS) (120). Pectins can be modified by pH and heat treatments, both of which expose galactoside residues by hydrolysis (139). The most studied modified pectin is MCP, which is obtained by partial degradation of citrus pectin polysaccharide chain and hydrolysis of the galacturonic acid esters from the HG regions. MCP through a multivalent display of galactoside residues has shown affinity for Gal-3 and has been tested as an anticancer agent in galectin-mediated tumorigenic processes (120).

The biological activity of MCP in cancer was extensively studied by Raz and coworkers (140–142). MCP has been shown to act as a ligand for Gal-3, while citrus pectin (CP) is unable to interact with this galectin (142). The authors showed that intravenous injection of MCP into mice bearing B16 melanoma resulted in significant decrease of lung colonization, while CP led to the opposite effect (142). In 2002, they expanded their findings to nude mice injected either with human breast carcinoma cells or with colon cancer cells, showing that the ability of MCP to inhibit primary tumor growth and metastasis *in vivo* is not restricted to prostate cancer (140). The antitumor effects of MCP were associated with an antiangiogenic activity, since MCP also inhibited capillary tube formation *in vitro* in human endothelial cells. Recently, Menachem et al. studied *in vivo* the combined inhibition of Ras and Gal-3 with FTS (*S*-*trans* farnesylthiosalicylic acid, Salirasib) and MCP, respectively, for the treatment of aggressive anaplastic thyroid carcinoma (143). Interestingly, FTS and MCP inhibited tumor growth in nude mice showing decreased levels of Gal-3, K-Ras-GTP, and p-ERK. However, the structure–function relationship of MCP and the molecular mechanisms underlying its effects are not yet completely understood, probably due to the lack of thorough structural characterization of pectin fractions. Nevertheless, MCP emerged as a very promising anticancer agent since it was the first reported inhibition of tumor growth by a soluble, orally ingested dietary carbohydrate fiber. In fact, two different commercial forms of MCP, PectaSol-C, and GCS-100, have been incorporated into clinical trials (112, 114, 115, 137, 138).

PectaSol (*23*, Table 3), and its most recent version Pectasol-C, commercial forms of MCP developed by EcoNugenics^®^ (Santa Rosa, CA, USA), showed cytotoxic activity on different prostate cancer cell lines (122, 123). Additionally, Jiang et al. demonstrated that the combination of PectaSol-C with two polybotanical compounds for breast and prostate health, BreastDefend (BD) and ProstaCaid (PC), synergistically inhibited metastatic phenotype of human breast and prostate cancer cell lines, respectively (121). In 2003, Guess and colleagues developed a Phase II clinical trial to investigate the tolerability and effect of PectaSol in patients with prostate cancer, showing that prostate cancer patients exhibited a serial increase in PSA after localized treatment (124). The results from this pilot clinical trial suggested that PectaSol may slow down PSA increase, as it improves prostate-specific antigen doubling time (PSADT) in patients with recurrent prostate cancer. However, PSADT is an indirect measure of tumor progression and the detailed effect that PectaSol may have on prostate cancer progression is still poorly understood. Currently, there is an ongoing patient recruitment by EcoNugenics for a Phase III clinical trial pointing at the effects of orally administered PectaSol-C for improving PSA kinetics in men with biochemical relapsed prostate cancer and serial increasing PSA (NCT: NCT01681823) (120).

GCS-100 (*24*, Table 3), another commercial MCP derivative, is also in clinical development for the treatment of cancer. GCS-100 inhibits cell growth in various multiple myeloma cell lines (126). Demotte et al. showed that GCS-100 influences the reinvigoration of anergic TILs: treatment with GCS-100 successfully detached Gal-3 from CD8^+^ TILs and boosted cytotoxicity and secretion of proinflammatory cytokines (144). Furthermore, in tumor-bearing mice vaccinated with a tumor antigen, injections of GCS-100 led to increased tumor rejection compared to control mice, showing the potential application of pectin-derived agents in combination with therapeutic vaccination as a combined cancer treatment (145).

GCS-100 was evaluated as an anticancer agent in three clinical trials, but two of them were suspended because of lack of funding (NCT00776802 and NCT00609817). The third one (La Jolla Pharmaceutical Company, NCT00514696) was a Phase II study of the safety of GCS-100 in 24 subjects with chronic lymphocytic leukemia. GCS-100 was delivered intravenously, showing excellent overall tolerability, partial remission in 25% of the patients and >50% shrinkage of lymph node lesions in 16% of patients (125, 146). Another interesting approach was reported by Zomer et al., who developed GR-MD-02 (*25*, Table 3), a pectin-derived galactoarabino-rhamnogalacturonan polysaccharide (127). GR-MD-02 is currently undergoing two clinical trials in combination with immune checkpoints inhibitors: the anti-PD-1 mAb Pembrolizumab and the anti-CTLA-4 mAb Ipilimumab for melanoma treatment (NCT02575404 and NCT02117362, respectively).

##### Galactomannan-Derived Compounds

Besides pectin-derived agents, β-d-(1 → 4)-galactomannan-based compounds, such as GM-CT-01 (trade name DAVANAT, *26*, Table 3), offer another alternative for galectin inhibition. GM-CT-01 is isolated from seeds of *Cyamopsis tetragonoloba* (Guar gum), and subjected to a controlled partial chemical degradation (147). With an average size of 51 kDa, this β-d-(1 → 4)-galactomannan is composed by galactose residues α(1 → 6)-linked to the mannose backbone at recurring intervals (148). GM-CT-01 has been mainly tested as an allosteric inhibitor of Gal-1 (*K*_D_ = 10 mM), but has also affinity for Gal-3, -7, and -9 (128, 149). GM-CT-01 has been evaluated alone or in combination with the chemotherapeutic agent 5-fluorouracil (5-FU) in preclinical studies. Phase I and II clinical studies for colon cancer treatment showed 70% higher stability for patients administered with GM-CT-01 and a 46% increase in survival of patients with end stage colon cancer (NCT00110721) (128). This galectin inhibitor also influenced human TILs from patients with various cancers, boosting IFN-γ secretion upon *ex vivo* stimulation in ~80% of CD8^+^ TILs and ~50% of CD4^+^ TILs (145). The response observed suggested that administration of GM-CT-01 may contribute to correct impaired TIL functions in cancer patients.

#### Peptides and Peptidomimetics

Peptides exhibit several advantages when compared to carbohydrate-based ligands. While carbohydrate–lectin interactions occur in the mid-micromolar range, peptide–protein or protein–protein interactions occur in the nanomolar range. Therefore, and since peptide synthesis has advanced considerably in the past few years, several research groups have developed an alternative approach using peptides or peptidomimetics to target galectins at their CRD or at distant sites.

In 2005, a pioneer work by Zou and colleagues reported binding of small peptides to Gal-3, with a concomitant reduction in cancer cell adhesion *in vitro* (150). Considering that Tf glycoantigen (Figure 1) is exposed on up to 90% of human carcinomas (81) and is a major cancer cell surface carbohydrate ligand for Gal-3, the authors obtained synthetic peptides mimicking the Tf structure using combinatorial bacteriophage display technology. The most promising compounds, G3-A9 (*27*, Table 3) and G3-C12 (*28*, Table 3), blocked interactions between Tf and Gal-3 and presented high affinity (72 nM) and good selectivity toward Gal-3 when compared to Gal-1, Gal-4, and LacNAc-binding plant lectins (123). These peptides effectively inhibited heterotopic adhesion of human breast carcinoma cells to endothelial cells, as well as homotypic tumor cell aggregation *in vitro*. Some years later, these authors also reported the ability of peptide G3-C12 (28) to modulate tumor establishment and growth of human breast carcinoma cells in mice, demonstrating a significant reduction of lung metastatic tumors (72%) by *in vivo* bioluminiscent imaging (129). G3-C12 (*28*, Table 3) was also studied in metastatic prostate cancer by Deutscher et al., who proposed radiolabeled G3-C12 as a marker of Gal-3-expressing prostate tumors (130). ^111^In-labeled G3-C12 peptide showed good tumor uptake in severe combined immunodeficient (SCID) mice bearing human prostate tumor xenografts. A major limiting factor for its application in prostate tumor imaging was its non-specific uptake, as it was also found in kidneys. Yang et al. solved this issue by coupling G3-C12 to water-soluble *N*-(2-hydroxypropyl)methacrilamide (HPMA) copolymers as drug carriers (151). These copolymers were further modified either with ^131^I radioisotope for *in vivo* SPECT-imaging (151), or with 5-FU for enhanced anticancer activity (152). ^131^I-G3-C12–HPMA copolymer showed higher tumor accumulation when compared to controls, although the authors experienced some difficulties with the adjustment of the ligand modification degree and the molecular size of the copolymer. On the other hand, 5-FU modified G3-C12–HPMA copolymer displayed a superior intracellular internalization followed by enhanced cytotoxicity and apoptosis induction in the PC-3 tumor-bearing mouse model (152). Recently, Sun et al. reported that the conjugation of G3-C12–HPMA copolymer with doxorubicin (G3-C12–HPMA–Dox) improved internalization into Gal-3-overexpressing PC-3 cells, but stimulated the translocation of Gal-3 to the mitochondria to prevent apoptosis (153). However, as time progressed, G3-C12–HPMA–Dox conjugates delivered increasing amounts of Dox into the mitochondria and overcame the anti-apoptotic effects of Gal-3. In spite of the lack of structural studies characterizing Gal-3/G3-C12 peptide binding, this peptide seems to be very promising not only as Gal-3-targeted imaging agent but also as therapeutic agent for Gal-3-overexpressing cancers such as melanoma and breast, ovarian, and gastric carcinomas (154).

Another example of peptide-based galectin ligand for cancer treatment is Anginex (*29*, Table 3), a 33-mer synthetic peptide originally designed to reproduce the β-sheet structure of antiangiogenic proteins like platelet factor 4 (PF4), interleukin (IL)-8, and bactericidal/permeability-increasing protein (BPI) (155, 156). This synthetic peptide has antiangiogenic and anti-tumor effects *in vitro* and *in vivo* (131, 157) and has been shown to bind Gal-1 (158), although it may also recognize other galectins such as Gal-2, -7, -8 (N-terminal), and -9 (N-terminal) (159). Later on, 6DBF7 (*30*, Table 3), a partial peptidomimetic of Anginex, bearing a hydrophobic dibenzofuran scaffold required for the β-sheet peptide configuration exhibited a better antiangiogenic performance in a mouse xenograft model of ovarian carcinoma (132).

In order to overcome the intrinsic susceptibility of peptides to hydrolysis by proteases, Dings et al. designed a non-peptidic topomimetic of Anginex and 6DBF7, based on a calixarene scaffold (134). This compound, named calixarene 0118 or OTX008 (*31*, Table 3), demonstrated potent angiogenesis inhibition in two mouse models of human ovarian carcinoma and murine melanoma. In addition, OTX008 has shown synergistic effects with sunitinib (a tyrosine kinase inhibitor) in ovarian carcinoma and glioblastoma mouse xenografts (133). Interestingly, OTX008 downregulates cancer cell proliferation, invasion, and tumor angiogenesis in a variety of tumor cells (160) and is undergoing a Phase I clinical trial by OncoTx Inc. (NCT01724320) (161).

## Conclusion and Future Perspectives

In the past few years, the field of cancer therapy has experienced an impressive breakthrough with the development of targeted therapies and immune checkpoint blockers (162). However, although significant improvements have been achieved with these therapeutic agents, some patients have not shown clinical benefits, presumably because of the development of adaptive resistance mechanisms and acquisition of compensatory pathways.

Aberrant glycosylation has emerged as a hallmark of cancer progression, and the presence of a tumor-associated glycome is deeply associated with malignant transformation and metastasis-associated processes including tumor cell migration, invasiveness, angiogenesis, and immune escape (14). However, the translational and clinical applications of altered glycan structures in cancer have not yet been completely accomplished, probably due to the complex regulation of the glycosylation machinery including glycosyltransferases and glycosidases. Heterogeneity is inherent to the language of glycans and crucial for their diverse biological roles as information carrier for lectins. On the other hand, lectins, glycan-binding receptors responsible for deciphering the glycome, arose as feasible targets for cancer therapy. As a result, considerable efforts have been made to disrupt glycan–lectin interactions by designing pharmacological anticancer agents that target different lectins including selectins, siglecs, and galectins. Further studies are still necessary to understand the precise underlying mechanisms of the antitumor effects displayed by lectin blockers, and to explore the potential complementation or synergy of turning-off lectin signaling with immune checkpoint blockade therapies, targeted therapies (e.g., small molecule inhibitors of receptor tyrosine kinases) and antiangiogenic therapies, as well as with chemotherapy, radiotherapy, and vaccination strategies. As of today, different approaches have generated chemical inhibitors for lectin–glycan interactions, resulting in diverse selectivity, affinity, and therapeutic efficacy in cancer models.

Small glycan derivatives and glycomimetics, which represent the majority of lectin blockers reported to date, target the carbohydrate-binding site and showed promising affinities and lectin selectivity *in vitro*. However, these carbohydrate-based ligands suffer certain disadvantages for their use in the clinics, i.e., low *in vivo* bioavailability, susceptibility to glycosidase hydrolysis, and fast clearance. For instance, for galectin inhibition, no galactose-based ligand has reached clinical trials up to date. Thus, alternative approaches have been made to circumvent these drawbacks including the design of hydrolytically stable N-, S-, and C-saccharides and the development of neoglycoproteins with higher bioavailability (163), but only a few of them have been successfully tested *in vivo*. Although not included in this review, metabolic inhibitors for sugar nucleotide biosynthesis were also proposed and efficiently tested: for example, per-acetylated 4-fluoro-glucosamine (4-F-GlcNAc) as a GlcNAc biosynthetic inhibitor was evaluated in cancer melanoma- or lymphoma-bearing mice, showing enhanced tumor lymphocytic infiltration and increased the frequency of cytotoxic T cells and IFN-γ production (164). However, these chemical inhibitors do not affect protein glycosylation in a specific way. Therefore, small carbohydrate inhibitors have to face diverse intrinsic drawbacks before reaching clinical settings.

Multivalent display by polysaccharide inhibitors has become an interesting approach for galectin and selectin inhibition. Heparin, dextran, and chitosan sulfated derivatives have been successfully tested as selectin inhibitors. Nevertheless, no detailed clinical trials have been yet designed to determine the antimetastatic effects of heparin derivatives in cancer. On the other hand, galactomannan-derived compounds, together with pectin-derived agents, are probably the most successful galectin chemical inhibitors that reached clinical evaluation in cancer patients. DAVANAT, GCS-100, and PectaSol currently being tested in clinical trials (among several other polysaccharides-based inhibitors) seem very promising anticancer agents, since they present high-binding affinities and can easily be obtained from natural sources. Moreover, these ligands stress the importance that dietary carbohydrate compounds may reach as therapeutic agents. Looking to the future, critical structure–function relationships and selectivity studies are still necessary, as well as elucidation of the precise mechanisms underlying interactions with these polysaccharide compounds. For example, the use of MCP as an anticancer agent is still limited because of lack of selectivity and structure variability (165). In fact, there are multiple MCPs on the market with different molecular weight range, diverse esterification degrees, and limited characterization at the molecular level. Although the affinity of MCPs for Gal-3 has been documented, there is still no study showing selectivity with regard to other lectins. In fact, MCP demonstrated the ability to detach cell surface binding of Gal-1 and Gal-3 to melanoma cells at comparable concentrations (144). In order to completely understand the biological modes of action of MCPs as well as other polysaccharide-based ligands in cancer-related processes, further studies are required to assess their affinity toward other lectins.

As alternative inhibitors, peptides and peptidomimetics have emerged as interesting agents capable of overcoming some of the intrinsic disadvantages of glycans. Among them, fluorinated C-manno-peptides represent promising therapeutic agents as selectin inhibitors, while G3-C12 (*28*) and the Anginex-derivative OTX008 (*31*) represent the most successful approaches for inhibition of Gal-3 and Gal-1, respectively; both are currently being tested in clinical trials.

With the advances in molecular biology and immunology, anti-sense nucleotides, mAbs, and lectin modifications have also been proposed as potential strategies for lectin inhibition. Small-interfering RNA (siRNA) has recently been offered as an alternative approach for galectin inhibition, but literature is still limited. In a recent study, Park et al. demonstrated that siRNA-mediated silencing of Gal-3 in a human osteosarcoma cell line resulted in decreased tumor cell migration and invasiveness (84). On the other hand, anti-E-selectin and anti-L-selectin mAbs have been successfully tested in the treatment of liver metastasis and ovarian cancer, respectively (49, 166, 167). In addition, modifications of natural selectin ligands such as P-selectin Glycoprotein Ligand-1 (PSGL-1) (i.e., by conjugation with IgG) afforded an auspicious selective inhibitor for interrupting leukocyte rolling and adhesion (168). In the case of galectin-inhibition, availability of recombinant proteins (i.e., truncated form of Gal-3) as well as mAbs (i.e., F8.G7 for Gal-1) opened new avenues for more specific and efficient blockers (34, 169–171). These therapies have been successfully tested *in vivo* and demonstrated a very good performance, suggesting their potential translation to clinical trials.

In summary, the data presented in this article stress the importance of understanding the biochemistry, biology, and glycobiology involved in tumor development and progression. The current major challenge faced by this field is designing selective inhibitors of lectin–glycan interactions with increased bioavailability. In turn, combined therapies of chemical inhibitors with biological therapeutics may tailor cancer treatments according to the unique features of individual tumors and patients.

## Author Contributions

AC and KM drafted the manuscript. AC and JS designed the figures and tables. All authors contributed with literature research, writing, discussion, and conclusions of the review. KM and GR performed a critical revision.

## Conflict of Interest Statement

The authors declare that the research was conducted in the absence of any commercial or financial relationships that could be construed as a potential conflict of interest.
